# Viral-host interactions mediated by the mTOR signaling pathway

**DOI:** 10.1016/j.cellin.2026.100311

**Published:** 2026-02-16

**Authors:** Zizhen Ming, Bing Su, Qiming Liang

**Affiliations:** a Center for Immune-Related Diseases at Shanghai Institute of Immunology, Ruijin Hospital, Shanghai Jiao Tong University School of Medicine, Shanghai, 200025, China; b Department of Immunology and Microbiology, Key Laboratory of Cell Differentiation and Apoptosis of Chinese Ministry of Education, Shanghai Jiao Tong University School of Medicine, Shanghai, 200025, China; c Department of Gastroenterology, Ruijin Hospital, Shanghai Jiao Tong University School of Medicine, Shanghai, 200025, China; d Shanghai Jiao Tong University School of Medicine-Yale Institute for Immune Metabolism, Shanghai Jiao Tong University School of Medicine, Shanghai, 200025, China

**Keywords:** mTOR signaling, Viral infection, Immune evasion, Autophagy, Antiviral therapy

## Abstract

The mechanistic target of rapamycin (mTOR) is an evolutionarily conserved serine/threonine kinase that regulates multiple key cellular processes. It assembles into two major multi-protein complexes, called mTOR complex (mTORC)1 and mTORC2, to integrate cues from cellular nutrient status, energy levels, and growth factors. mTOR plays crucial roles in key physiological processes such as protein synthesis, autophagy initiation, lipid metabolism, and cell survival. As obligate intracellular parasites, viruses rely heavily on the host's biosynthetic machinery, making viral propagation dependent on host-derived metabolic resources. Consequently, the metabolic networks and cellular functions governed by the mTOR pathway directly support the viral life cycle, establishing it as a critical regulatory node in virus-host interactions. To fulfil their replication demands, viruses have evolved diverse strategies to manipulate the mTOR signaling: sustained activation of host anabolic metabolism, selectively inhibition the mTOR-mediated autophagy to generate membranous structures, and dynamically tuning mTORC1 and mTORC2 activities to meet stage-specific replication needs. This review systematically elucidates the structural basis and regulatory landscape of the mTOR signaling pathway, highlighting the specific mechanisms used by various viruses to modulate the mTOR function. It also examines the central role of mTOR in antiviral immunity and provides preclinical and clinical evidence supporting mTOR-targeted antiviral strategies. Ultimately, this review aims to outline a comprehensive theoretical framework for understanding virus-host interactions through mTOR modulation and offers novel perspectives on the development of mTOR-based antiviral interventions.

## 1 Introduction

The metabolic state and physiological resilience of the host are critical determinants of whether a virus can establish a productive infection. Following infection, the competition between pathogens and the host for limited cellular resources becomes a pivotal factor influencing viral replication and host defense mechanisms (Virgin et al., 2009; Thaker et al., 2019). Within this interaction, the regulation of core host signaling pathways serves as a central battleground, with the mTOR signaling pathway emerging as a key target for viruses due to its role as a “metabolic master switch” (Saxton and Sabatini, 2017). The mTOR signaling responds to external cues, including nutrients, energy sources, and growth factors, to maintain a dynamic balance between cellular anabolic and catabolic processes (Fernandes et al., 2024; Goul et al., 2023; Hay and Sonenberg, 2004; Laplante and Sabatini, 2012; Ortega-Molina et al., 2024; Wu et al., 2021; Yong et al., 2025). By promoting protein and lipid synthesis while suppressing autophagy, mTOR creates a pro-anabolic environment that provides the structural components and biosynthetic capacity essential for efficient viral replication (Panwar et al., 2023).

Ongoing research at the intersection of virology and immunometabolism continues to highlight the complexity and diversity of interactions between viruses and the host mTOR signaling pathway (Xie and Proud, 2014). Certain viruses enhance host translational capacity and metabolic flux by activating the mTOR pathway, thereby securing resources essential for viral genome replication and particle assembly (Chuluunbaatar et al., 2010; Walsh and Mohr, 2011). Others strategically inhibit mTOR signaling to induce autophagy, exploiting autophagic membranes as scaffolds for replication complexes (Liang et al., 2016). Additionally, some viruses have evolved temporal, bidirectional strategies to modulate mTOR activity during different stages of infection, thereby optimizing replication efficiency (Kuss-Duerkop et al., 2017; Sharma et al., 2020). Recent research on globally prevalent viruses, such as Hepatitis B virus (HBV) and Severe Acute Respiratory Syndrome Coronavirus-2 (SARS-CoV-2), has gradually unveiled critical mechanisms of their interaction with the mTOR pathway, reinforcing its central role in both chronic and emerging viral infections (Guarnieri et al., 2023; Mei et al., 2024).

Notably, beyond its role in metabolic regulation, the mTOR signaling is integral to immune homeostasis, governing innate immune activation (Weichhart et al., 2015), adaptive immune cell differentiation (Jones and Pearce, 2017), and immune tolerance (Cobbold, 2013). The mTORC1 and mTORC2 coordinately govern T cell fate decisions by integrating antigenic, co-stimulatory, cytokines, and metabolic signals (Pollizzi et al., 2015; Rosenlehner et al., 2024). During immune activation, mTOR signaling promotes the effector differentiation of CD4^+^ and CD8^+^ T cells while suppressing regulatory and memory programs, functioning as a central hub linking antigen receptor signaling, cytokines, and metabolic pathways (Chen et al., 2023a; Wang et al., 2022a). Given its dual role in shaping immunity and tolerance, mTOR inhibition induces immunosuppression in the contexts such as transplantation and autoimmunity, yet can also be leveraged to enhance vaccine efficacy or modulate antitumor immunity (Chi, 2012; Joo et al., 2024; Withers et al., 2025). Consequently, viral modulation of mTOR signaling not only reprograms host cellular metabolism but also remodels antiviral immune responses, thereby facilitating immune evasion (Moreno-Altamirano et al., 2019; Li et al., 2023; Darweesh et al., 2025).

Although substantial progress has been made in uncovering virus–mTOR interactions, several critical mechanistic questions remain unresolved. These include the distinct and context-dependent functional specificity of mTORC1 and mTORC2 in viral infection (Kuss-Duerkop et al., 2017), the incomplete mechanistic understanding regarding major pathogens like SARS-CoV-2 (Camps et al., 2025) and less-studied viruses such as Ortho Flaviviruses (Zhang et al., 2025), and the poorly defined crosstalk between immunometabolism networks and mTOR signaling axis (Liu et al., 2017a). Furthermore, although mTOR-targeted antiviral therapy demonstrates considerable promise, its clinical application remains challenging due to the pathway's complexity, systemic adverse effects, and the lack of clarity distinguishing preclinical from clinical evidence (Kaplan et al., 2014; Maiese, 2020; Mullen et al., 2021). In light of these challenges, this review provides a systematic overview of the structural basis of the mTOR signaling pathway, the diverse regulatory strategies employed by viruses to modulate this pathway, mTOR-mediated antiviral immune mechanisms, and the hierarchy of evidence for targeted therapies. This review aims to offer a comprehensive perspective for advancing research in this field and to provide theoretical guidance for the development of novel antiviral interventions.

## 2 Overview of the mTOR signaling pathway

Functioning as a central coordinator of cellular signaling, mTOR assembles into two major complexes: mTORC1 and mTORC2 (Battaglioni et al., 2022; Laplante and Sabatini, 2012; Linke et al., 2017). Each complex exhibits distinct structural compositions and functional roles (Virgin et al., 2009; Yang et al., 2013; Goul et al., 2023). mTORC1 is a protein kinase complex composed of mTOR, raptor, mLST8/GβL, and DEPTOR (Aylett et al., 2016; Jacinto et al., 2006). Its activation is finely regulated by multiple upstream signals, for instance, amino acids activating mTORC1 via a lysosome-targeting mechanism dependent on Rag and Rheb GTPases (Sancak et al., 2008; Yang et al., 2017). Growth factors signal through the PI3K-AKT-TSC axis, where AKT phosphorylation suppresses the TSC1/2 complex, thereby relieving its inhibitory effect on mTORC1 and enhancing mTOR signaling (Dibble and Cantley, 2015; Inoki et al., 2002). Under low-energy conditions, mTORC1 activity is dampened via phosphorylation of Raptor or the enhancement of TSC2 (Gwinn et al., 2008; Saxton and Sabatini, 2017). Activation of mTORC1 broadly coordinates anabolic growth program by enhancing cap-dependent protein synthesis and ribosome biogenesis while concurrently repressing autophagy initiation, thereby prioritizing biosynthetic processes over catabolic recycling pathways (He et al., 2024; Hosokawa et al., 2009; Böhm et al., 2021; Battaglioni et al., 2022; Hajdú et al., 2022).

**Fig. 1:**
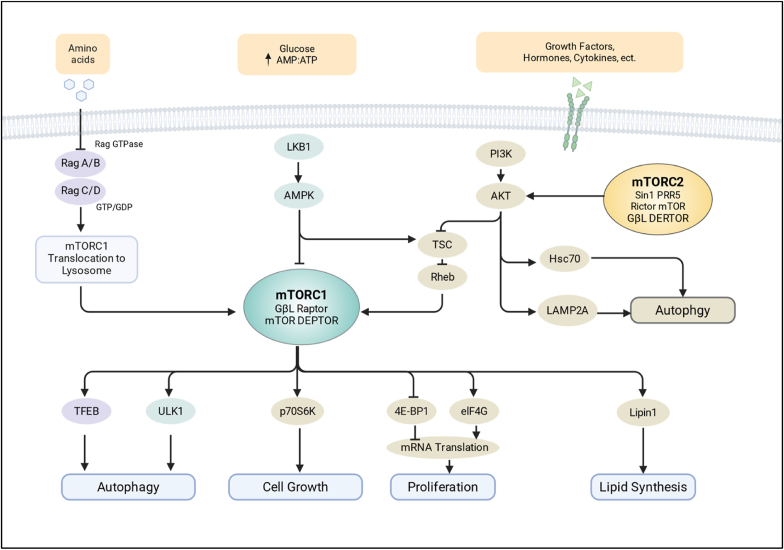
**Schematic diagram of the structure, upstream regulatory mechanisms, and downstream biological functions of mTORC1 and mTORC2**. The figure shows the structural framework of the mTOR signaling system, including the core components of the two functional complexes: mTORC1 (mTOR, Raptor, DEPTOR, mLST8) and mTORC2 (mTOR, Sin1, PRR5, Rictor, mLST8); upstream regulatory pathways include the PI3K–AKT–TSC–Rheb axis (growth factors, hormones, and cytokines), the amino acid–Rag GTPases–lysosomal localization pathway (nutrient signals), and the AMPK–energy stress pathway (energy signals); downstream effector pathways include classic modules such as protein translation (S6K1, 4E-BP1), lipid metabolism, and autophagy inhibition (ULK1).

The mTORC2, which consists of mTOR, Rictor, mSIN1, PRR5, and mLST8 (Chen et al., 2018; Khan et al., 2015; Taylor et al., 2025), is primarily activated by growth factor signaling rather than nutrient cues (Fu and Hall, 2020; Palma et al., 2023; Suzuki and Inoki, 2011). mTORC2 regulates cell survival, metabolism, and cytoskeletal organization largely via the phosphorylation of AKT at Ser473 (Saxton and Sabatini, 2017; Taylor et al., 2025). Recent studies have expanded the functional scope of mTORC2, highlighting roles in metabolic remodeling and stress-responsive signaling networks relevant to both cancer biology and viral infection contexts. mTORC2 has been implicated in the regulation of cellular metabolic programs such as gluconeogenesis and proliferative capacity, while also facilitating ligand-induced activation of IGF-IR/InsR signaling through SIN1-mediated interactions with IRS (Khan et al., 2015; Yin et al., 2016). In parallel, mTORC2 modulates actin cytoskeleton dynamics and cell polarity via small GTPase signaling pathways, thereby influencing processes such as cell migration and phagocytic activity (Yang et al., 2023). Inhibition of mTOR kinase activity further triggers compensatory autophagic responses and suppresses cell motility, underscoring the multifaceted roles of mTORC2 in cellular stress adaptation and structural remodeling (Chen et al., 2012). Fig. 1 provides an overview of this signaling network, detailing the components of mTORC1 and mTORC2, their major upstream activation pathways, and the diverse downstream biological processes they regulate.

The mTOR signaling network integrates various extracellular and intracellular signals through upstream sensing modules (Hay and Sonenberg, 2004; Laplante and Sabatini, 2012; Yang et al., 2013), governing essential processes such as protein and lipid synthesis, mitochondrial function, and autophagy (Saxton and Sabatini, 2017; Shimobayashi and Hall, 2014). The PI3K-AKT-mTOR axis represents one of the primary targets hijacked by viruses during infection (Soares et al., 2009; Yin et al., 2018). Viruses engage different nodes of this pathway, including receptor-mediated activation of PI3K by vaccinia virus (VACV) and cowpox virus (CPXV) during early infection (Soares et al., 2009); inhibition of AKT signaling by vesicular stomatitis virus (VSV) (Dunn and Connor, 2011) and Rift Valley fever virus (RVFV) (Popova et al., 2010) to attenuate mTOR activity; and the exploitation of pre-S1-VEGFR-2 and HBx-PI3K interactions by HBV to activate the pathway (Wang et al., 2013a).

Importantly, viral manipulation of mTOR signaling is often dynamic and stage-dependent, involving coordinated activation and suppression to optimize replication while evading host immune responses. During early phases of infection, many viruses activate mTOR to enhance cap-dependent translation and viral protein synthesis. For instance, Influenza A virus (IAV) exploits early PI3K–Akt–mTOR axis to facilitate early step of viral entry, despite its role in triggering IRF-3 mediated antiviral defenses (Ehrhardt et al., 2006). Herpes simplex virus-1 (HSV-1) constitutively activates mTORC1 shortly after entry to suppress apoptosis and to support viral translation (Benetti and Roizman, 2006), whereas SARS-CoV-2 engages mTORC1 through viral proteins such as Nsp14 to stimulate host translational and metabolic programs (Zhou et al., 2025). Similarly, Human Cytomegalovirus (HCMV) activates and maintains mTOR signaling very early after infection by re-localizing mTORC1 to a perinuclear, Rheb-associated compartment, enabling sustained activity even under cellular stress to support viral replication (Clippinger and Alwine, 2012; Clippinger et al., 2011a). This early mTOR activation allows viruses to efficiently exploit host biosynthetic and metabolic resources essential for productive replication.

At later stages of infection or under heightened immune pressure, certain viruses shift toward suppression or functional dysregulation of mTOR signaling to attenuate host antiviral defenses and, in some cases, promote viral persistence or latency. This fact is often accompanied by induction of autophagy, dampening type I interferon (IFN) signaling, suppression of interferon-stimulated genes (ISGs), or evasion of cytosolic sensing pathways. For instance, during established infections, Hepatitis C virus (HCV) inhibits the AKT-TSC-mTORC1 signaling through ER stress, leading to ULK1 activation and induction of autophagy that supports replication organelle (DMV) formation and viral persistence (Huang et al., 2013). Similarly, SARS-CoV-2 utilizes ORF7a to inhibit mTOR signaling and disrupt autophagic flux, thus creating an autophagy environment that facilitates efficient viral propagation (Hou et al., 2023). Moreover, during late-stage poxvirus replication, the viral protein F17 localizes to mitochondria and dysregulates mTOR signaling, destabilizing cGAS and suppressing glycolysis to attenuate antiviral immunity and facilitate continued viral replication (Meade et al., 2023).

Given that mTOR-regulated processes are involved in nearly every stage of viral infection*—*from nucleotide and protein synthesis to membrane formation and cell survival (Crater et al., 2022)*—*the mTOR pathway represents a critical molecular interface in virus–host interactions (Jiao et al., 2025; Wei et al., 2024). The following sections will detail the specific strategies pathogens use to exploit mTOR pathway for efficient replication and assembly.

## 3 Viruses use the host mTOR pathway for replication

**Fig. 2:**
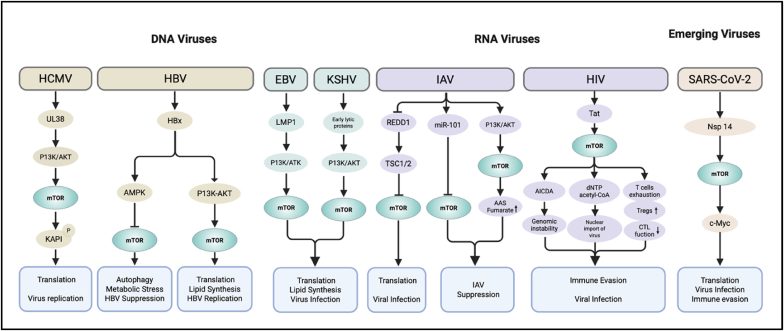
**Multiple viruses activate the mTOR signaling pathway through different molecular mechanisms to promote replication.** The figure presents the viral strategies that converge on mTOR activation across DNA viruses (HCMV, HBV, KSHV, EBV), RNA viruses (IAV, HIV), and emerging viruses (SARS-CoV-2). (1) HCMV sustains mTORC1 activity through the viral protein UL38, primarily by relieving TSC2-mediated repression, whereas HBV engages the PI3K–AKT pathway and exhibits context-dependent regulation via AMPK-associated and PI3K/AKT signaling; (2) The γ-herpesviruses EBV and KSHV activate mTOR signaling through viral oncogenic proteins such as LMP1 or early/lytic proteins that converge on upstream PI3K/AKT nodes; (3) Among RNA viruses, IAV downregulates REDD1 to relieve TSC1/2-dependent inhibition of mTORC1, while virus-induced miR-101 upregulation provides a counter-regulatory brake on mTOR activity; HIV Tat promotes mTOR activation; (4) SARS-CoV-2 nsp14 interacts with Raptor to enhance mTORC1 signaling. Activated mTOR reprograms host metabolism by enhancing cap-dependent translation, lipid biosynthesis, and nucleotide production, thereby supporting viral genome replication, virion assembly, and infected-cell survival.

The primary objective of viruses activating the mTOR pathway is to remodel the cellular metabolism and biosynthetic capacity, thereby creating a permissive environment for efficient viral replication. Although molecular strategies vary among viruses, their mechanisms frequently converge on central nodes of the mTOR network, particularly mTORC1, to enhance protein translation, lipid synthesis, and nucleotide production required for viral genome replication and virion assembly. To provide a comprehensive overview, Fig. 2 illustrates the cellular targets within the mTOR pathway, and the activation strategies employed by various pathogens, including HCMV, Hepatitis B Virus (HBV), Epstein-Barr virus (EBV), Kaposi's sarcoma-associated herpesvirus (KSHV), IAV, Human immunodeficiency virus (HIV), and SARS-CoV-2.

### 3.1 HCMV and HBV

HCMV extensively modulates the mTOR pathway to create a cellular environment permissive for viral replication (Bie et al., 2025; Miller et al., 2024). Activation of mTOR signaling promotes viral gene expression and productive infection by enhancing host anabolic metabolism and cap-dependent translation, thereby supplying essential substrates for viral protein synthesis and virion assembly (Laplante and Sabatini, 2012; Park et al., 2025). In parallel, HCMV reprograms the autophagic processes through viral proteins such as US12, altering ULK1 activity, conversion of LC3, and SQSTM1/p62 turnover to fine-tune autophagic flux in support of viral persistence and replication (Liu et al., 2017b; Altman et al., 2020; Kim et al., 2023; Krämer et al., 2024).

Similarly, Hepatitis B Virus (HBV) replication is regulated by multiple host signaling pathways, notably the PI3K–AKT–mTOR axis (Xiang and Wang, 2018). The viral HBx protein simultaneously activates both AMP-activated protein kinase (AMPK) and mTORC1 pathways, which exert opposing inhibitory and promotive effects on HBV viral replication, respectively. This dual engagement positions HBx as a “molecular rheostat” that dynamically balances metabolic stress responses and biosynthetic signaling to optimize HBV replication efficiency (Bagga et al., 2016). Beyond viral replication, HBx-mediated mTOR modulation has also been linked to broader pathological outcomes in extrahepatic tissues (Ai et al., 2023).

### 3.2 EBV and KSHV

EBV and KSHV, both members of the γ-Herpesvirus subfamily, utilize long-term latency as their primary survival strategy. EBV establishes lifelong latency in memory B cells and modulates viral gene expression to evade host immune surveillance, a process contributing to lymphomas, nasopharyngeal carcinoma (NPC), and other malignancies (Dugan et al., 2019). Mechanistically, particularly during lytic reactivation, EBV activates the mTOR signaling pathway and increases the phosphorylation of downstream substrates, including S6K1 and 4E-BP1, thereby creating a cellular environment favorable for viral DNA replication and virion assembly (Huang et al., 2017). Additionally, the EBV-encoded latent membrane protein 1 (LMP1) serves as a key viral oncogene implicated in this tumorigenesis (Wang and Ning, 2021).

Similarly, KSHV establishes long-term latency in B cells and endothelial cells, causing persistent infection in primates (Shifflett and Dittmer, 2025). KSHV-infected human endothelial cells exhibit activated mTORC1 signaling, rendering them sensitive to pharmacological mTOR inhibition (Das et al., 2023). During the lytic phase, early lytic proteins of KSHV stimulate or mimic mTORC1 activation to exploit host translational machinery. Although some studies suggest that mTORC1 activity may be dispensable for viral protein synthesis per se, KSHV lytic replication disrupts mTORC1-mediated autophagy regulation. Collectively, these findings indicate that KSHV subverts mTORC1-dependent cellular processes during the lytic cycle to ensure efficient viral replication (Pringle et al., 2019).

### 3.3 IAV and HIV

RNA viruses frequently employ host mTOR signaling to modulate cellular metabolism and immune responses. This manipulation is notably dynamic, often involving time-dependent bidirectional control and multidimensional interventions to balance replication efficiency with host cell survival. Such adaptability aligns with the intrinsic characteristics of RNA viruses, such as short replication cycles and a high propensity to trigger host immune responses (Palmer, 2022; Zhang et al., 2025).

In IAV infection, mTOR signaling exhibits a phase-specific pattern characterized by early activation followed by subsequent suppression under specific cellular contexts. During the early phases of infection, the suppression of REDD1 elevates mTORC1, enabling the host translational machinery to prioritize viral protein synthesis and accelerate replication-complex assembly (Kuss-Duerkop et al., 2017). Furthermore, IAV infection remodels host cellular metabolism via coordinated activation of PI3K/AKT/mTORC1 signaling. This promotes a shift toward aerobic glycolysis, creating an optimal environment that supports RNA replication and protein synthesis (Hui et al., 2025). In immune cells such as macrophages, IAV drives mTORC1 activation to promote sustained inflammation and metabolic remodeling rather than immediate viral replication (Huckestein et al., 2024). Specifically, IAV-associated activation of mTOR signaling promotes glycolytic remodeling that facilitates viral RNA and protein synthesis, whereas the host counteracts this by activating the aspartate–argininosuccinate (AAS) shunt, increasing fumarate via ASS1 as part of an antiviral metabolic defense program (Ren et al., 2021; Xia et al., 2025). As infection progresses, host regulatory mechanisms such as miRNA-mediated mTOR suppression emerge, providing negative feedback to limit viral replication and cellular stress (Diggins and Hancock, 2023; Sharma et al., 2020).

Similarly, HIV modulates mTOR pathway through multiple mechanisms to support viral replication, facilitate immune evasion, and contribute to associated pathologies (Crater et al., 2022). The HIV-1 proteins such as Tat activates the AKT-mTORC1 pathway to promote aberrant gene expression and genomic instability in infected cells, contributing to oncogenic transformation of B-cell lymphoma in HIV-infected individuals (Akbay et al., 2021). Chronic HIV infection is further characterized by mTOR-influenced immune dysregulation, including T cell exhaustion, expansion of regulatory T cells (Tregs), and impaired cytotoxic T lymphocyte function, collectively establishing an immunosuppressive environment that severely compromises viral clearance (Taylor et al., 2018; Fenwick et al., 2019). Beyond immune modulation, mTOR-driven metabolic reprogramming supplies critical resources for HIV replication, including nucleotide pools for reverse transcription and acetyl-CoA for efficient nuclear import of viral cDNA, which is essential for establishing efficient infection (Taylor et al., 2020). This metabolic reprogramming underscores how HIV co-opts this central host pathway to enhance viral fitness, a defining feature of chronic HIV infection (Crater et al., 2022). Emerging evidence further indicates that HIV-1 replication and latency are tightly regulated by mTOR-driven metabolism, with mTOR functioning as a molecular switch that dictates whether infected cells support active replication or enter latency, positioning it as a critical target for HIV cure strategies (Crater et al., 2022).

### 3.4 SARS-CoV-2

SARS-CoV-2 hijacks mTOR pathway to integrate metabolic reprogramming with immune invasion, driving robust viral replication and pathogenesis (Mullen et al., 2021; Stukalov et al., 2021). Following infection, the viral non-structural proteins such as Nsp14 activates the mTORC1 signaling in a Raptor-dependent manner to facilitate viral replication. However, the precise mechanism, such as direct interactions between Nsp14 and Raptor, or regulatory connections with the TSC1/2 complex, remains to be experimentally validated (Zhou et al., 2025). Furthermore, mTORC1 activation has been associated with induced expression of SAM synthase (MAT2A) via phosphorylation of the transcription factor c-Myc. MAT2A catalyzes the production of SAM, the primary methyl donor required for m^6^A modification of host and viral mRNA. Consequently, this signaling cascade markedly elevates global m^6^A modification levels across cellular and viral transcriptomes (Villa et al., 2021; Zhou et al., 2025). Functionally, m^6^A modification of specific SARS-CoV-2 RNA regions, such as ORF1ab, significantly enhances translational efficiency, accelerates the synthesis of non-structural proteins required for replication–transcription complex (RTC) assembly, and promotes genome replication. Conversely, m^6^A modification of host immune-related transcripts, such as interferon-β (IFN-β) mRNA, reduces their stability by promoting degradation or inhibiting translation, thereby dampening innate immune responses (Burgess et al., 2021; Winkler et al., 2019; Zhang et al., 2021). Pharmacological targeting of the m^6^A RNA modification pathway has been shown to effectively block SARS-CoV-2 and hCoV-OC43 replication (Burgess et al., 2021). Given the crosstalk between mTOR signaling and RNA modification, this further highlights the therapeutic potential of interfering these interconnected host dependencies (Laplante and Sabatini, 2012). Collectively, the mTORC1-m^6^A axis emerges as a key mechanism by which SARS-CoV-2 rapidly replicates and evades immune clearance.

Collectively, viruses across distinct families strategically hijack mTOR signaling to coordinate metabolic remodeling, protein synthesis, and cellular survival in favor of replication. This manipulation is often highly dynamic and context-dependent, underscoring mTOR as a central regulator exploited throughout different stages of viral infection.

## 4 Viral reprogramming of the mTOR signaling to circumvent host immune defenses

Unlike viruses that upregulate the mTOR pathway to commandeer metabolic resources, many pathogens reprogram the mTOR pathway to attenuate host immune defenses. Rather than uniformly suppressing mTOR activity, viruses selectively modulate distinct nodes within the mTOR network, including mTORC1-dependent translational control, autophagy checkpoints, and mTOR-regulated immune signaling pathways, to reshape the cellular environment in their favor. This complex manipulation occurs across distinct mechanistic layers, each targeting a specific vulnerability in the host cellular defenses.

### 4.1 Control of host translation machinery

The mTOR signaling controls ribosome recruitment and general protein synthesis by activating its two major effectors: S6K1 and 4E-BP1. Activation of mTORC1 promotes cap-dependent translation by relieving 4E-BP1-mediated repression of the eIF4F initiation complex and enhancing ribosome biogenesis and translational efficiency via S6K1 signaling (Ma and Blenis, 2009). However, upstream modulators can fine-tune mTORC1 output, selectively shaping translational programs without uniformly activating all downstream branches, as exemplified by regulatory mechanisms that preferentially suppress 4E-BP1 phosphorylation while sparing S6K1 activity (Wu et al., 2018).

Viruses mimic or exploit these regulatory mechanisms to modulate protein synthesis, subverting host translation by reprograming mTOR/4E-BP1 axis to outcompete the host's finite ribosomal capacity (Walsh and Mohr, 2006, 2011; Clippinger et al., 2011b; Yin et al., 2018; Wang et al., 2022a). For instance, in porcine reproductive and respiratory syndrome virus (PRRSV) infection, nsp2-derived viral proteins disrupt the mTOR signaling cascade and halt host protein synthesis, illustrating a targeted strategy to interfere with host translation control (Jiao et al., 2025). Negative-strand RNA viruses (e.g. VSV and IAV) induce host translation shutoff via distinct mechanisms (Walsh and Mohr, 2011; Walsh et al., 2013). VSV primarily targets the mTORC1/4E-BP1 axis. Its matrix (M) protein blocks AKT activation, which suppresses mTORC1 activity and maintains 4E-BP1 in an active and dephosphorylated state. This allows disruption of the eIF4F initiation complex and selectively blocking cap-dependent translation of host mRNAs while favoring viral protein synthesis (Ahmed et al., 2024; Connor and Lyles, 2002; Dunn and Connor, 2011; Montero et al., 2015). In contrast, IAV uses its NS1 protein to sequester or inhibit PKR, thereby preventing eIF2α phosphorylation and enabling controlled regulation of host translation to support viral replication (Walsh et al., 2013; Ji et al., 2021). Host defenses, such as APOBEC3B-driven PKR activation leading to translation shutdown and stress granule formation, can counter viral spread (Manjunath et al., 2023).

Additionally, viral proteases (e.g. enteroviruses and rhinoviruses) cleave eIF4G-I, thus resulting in a truncated fragment that abolishes cap recognition. Consequently, utilizing the internal ribosome entry site (IRES) is crucial for paramyxoviruses to achieve translation independence, allowing them to remain unaffected by the cap-binding complex (Francisco-Velilla et al., 2022; Lloyd, 2006). These IRES elements recruit ribosomes directly to facilitate cap-independent translation (Yang and Wang, 2019; Waheed et al., 2023). Furthermore, flaviviruses such as HCV and dengue virus (DENV) employ a distinct strategy to suppress host translation while preserving viral protein synthesis. HCV activates mTORC1 through NS5A–eIF4F interaction, maintaining 4E-BP1 phosphorylation (George et al., 2012). In contrast, DENV inhibits host interferon mRNA translation via sfRNA-mediated sequestration of RNA-binding proteins (G3BP1, G3BP2, CAPRIN1) (Bidet et al., 2014) and p38–Mnk1-driven eIF4E phosphorylation (Roth et al., 2017). Both viruses sustain IRES-dependent translation, which remains active under conditions of suppressed cap-dependent initiation, thereby circumventing 4E-BP1-mediated inhibition through alternative initiation mechanisms (Fernández-García et al., 2021).

Overall, viral reprogramming of mTOR-centered translational control enables selective host shutoff while preserving efficient viral protein synthesis, representing a conserved strategy for maximizing replicative fitness across diverse viral families.

### 4.2 Autophagy modulation

As a crucial cellular defense mechanism, autophagy is intricately linked with mTOR pathway and viral infection. These modulatory strategies represent evolved viral adaptations that synchronize host pathway modulation with viral replication kinetics, allowing viruses to subvert autophagy for replication while simultaneously attenuating antiviral immunity (Chen et al., 2023a). Rather than uniformly activating canonical autophagy cascades, diverse viruses employ distinct strategies to reprogram autophagic machinery through mTOR-dependent mechanisms. While some viruses inhibit or evade autophagic processes, others hijack the pathway, exploiting autophagic membrane structures as replication scaffolds or using the mechanism to circumvent host immune defenses (Chen et al., 2023a). For instance, Echovirus, a human enterovirus, induces autophagy through bidirectional reprogramming of key autophagy molecules*—*inhibiting mTOR and ULK1 phosphorylation while activating Vps34 and Beclin-1*—*rather than through a simple bypass mechanism (Wu et al., 2023). In contrast, Coxsackievirus B3 (CVB3) triggers a non-standard autophagy process through bypassing the reliance on the ULK1 and PI3K-dependent initiation pathways (Mohamud et al., 2020). To construct a replication scaffold, CVB3 utilizes protease 3C to cleave ULK1, while concurrently relying on PI4KIIIβ to drive membrane formation. Comparing the mTORC1 inhibitor rapamycin and the PI3K inhibitor ZSTK474 demonstrates that the timing of autophagy inhibition is critical for regulating viral replication (Chang et al., 2018). Notably, extracellular vesicles derived from cardiac progenitor cells can alleviate CVB3-induced apoptosis via regulation of the mTOR/S6K1/4E-BP1 axis (Li et al., 2019b), highlighting the therapeutic potential of targeting these pathways (Garmaroudi et al., 2015).

Several RNA viruses further exploit metabolic autophagy programs through coordinated AMPK activation and mTORC1 inhibition. DENV activates AMPK while concurrently suppressing mTORC1, triggering selective autophagy that degrades lipid droplets to fuel β-oxidation. Both AMPK signaling and mTORC1 inhibition are essential for inducing lipophagy and supporting robust DENV replication, revealing a dual regulatory mechanism for viral exploitation of host lipid metabolism (Jordan and Randall, 2017). Similarly, chikungunya virus (CHIKV) replication activates the IRE1α–XBP1 arm of the unfolded protein response, triggering autophagy (Joubert et al., 2012). In parallel, CHIKV-induced ROS/RNS stimulate AMPK, which phosphorylates TSC2 and Raptor to inhibit mTORC1 (Gwinn et al., 2008; Joubert et al., 2015). This inhibition restores ULK1 activity, promoting autophagosome formation to support viral replication and delay apoptosis (Joubert et al., 2012). HCV manipulates this pathway through a specific sequence of events: infection induces endoplasmic reticulum (ER) stress, which subsequently inhibits the AKT signaling axis. This inhibition of AKT leads to the suppression of mTORC1, relieving the negative regulation on autophagy and thereby upregulating lipid degradation to fuel the viral life cycle (Huang et al., 2013). These findings demonstrate that viruses dynamically manipulate mTOR-regulated autophagy to remodel intracellular metabolism and survival pathways, establishing autophagy as a key platform supporting efficient viral replication.

### 4.3 Immune evasion induced by viral inhibition of mTOR

Viruses have evolved diverse mechanisms to evade host immune responses. Beyond inducing autophagy, inhibiting the mTOR pathway represents a critical strategy for evading immune surveillance (Alghamdi et al., 2025). Viruses deploy encoded proteins to interfere with mTOR pathway, thereby disrupting host immune-related protein synthesis, impairing antiviral IFN signaling, and degrading antiviral molecules (Alghamdi et al., 2025).

As a central regulator of both innate and adaptive immunity, mTOR activity directly governs IFN production, antigen presentation, and immune cell function (Nouwen and Everts, 2020; Alghamdi et al., 2025). Pathogens frequently manipulate this pathway in macrophages and dendritic cells (DCs), key myeloid cells of the innate immune system, to subvert immune responses and promote viral survival (Nouwen and Everts, 2020). For instance, HSV-1 employs UL41 to inhibit mTOR in macrophages, resulting in host translation shutoff and reduced pro-inflammatory cytokine production (IL-1β, IL-8, MIP-1) (Suzutani et al., 2000). HCV upregulates arginase 1 in CD33^+^ PBMCs, depleting L-arginine and inhibiting mTOR signaling, which suppresses IFN-γ production by co-cultured NK cells (Goh et al., 2016). Moreover, in hepatocytes, chronic pathogens such as the HCV inhibit the AKT-TSC-mTOR axis by inducing ER stress (Huang et al., 2013). This inhibition compromises the maturation of these antigen-presenting cells (APCs), specifically impairing their capacity to process and present viral antigens to T cells. Consequently, mTOR suppression dampens the production of pro-inflammatory cytokines and IFNs, thereby weakening the host's early antiviral defenses (Nouwen and Everts, 2020).

Viruses interact with the mTOR pathway to regulate replication, IFN responses, and host translation. mTORC1, the key complex governing cell growth and metabolism, plays a pivotal role in shaping innate immunity by regulating the antigen-presenting capabilities of DCs and the phagocytic functions of macrophages. Consequently, host cells rely on mTORC1 and autophagy to inhibit viral replication and latency (Weichhart et al., 2015). However, viruses have evolved specific countermeasures. For instance, HIV interferes with mTORC1 at all stages of infection (Akbay et al., 2020), suggesting that targeting this complex offers a promising strategy for HIV-1 eradication and management of HIV-related diseases. Similarly, Porcine Reproductive and Respiratory Syndrome Virus (PRRSV) differentially regulates mTOR pathway genes in porcine alveolar macrophages; here, mTOR controls infection by modulating type I IFN production, forming a bidirectional loop with the IFN system. Viruses also manipulate mTOR to control translation (Liu et al., 2017a). Newcastle Disease Virus (NDV) activates PI3K/Akt/mTOR and p38 MAPK/Mnk1 pathways to enhance host cap-dependent translation. Even when mTOR is inhibited, p38 MAPK/Mnk1 axis mediates 4E-BP1 hyperphosphorylation to sustain viral protein synthesis, while the NDV NP protein binds eIF4E to enable selective viral mRNA translation (Zhan et al., 2020). Conversely, Canine Parvovirus (CPV-2) non-structural protein 1 (NS1) induces host translation shutoff by reducing mTOR phosphorylation, a mechanism unrelated to transcription and protein degradation, thereby facilitating immune evasion and viral replication (Wang et al., 2025).

Additionally, some viruses influence the differentiation and fate of T cell subsets, including effector, regulatory, follicular helper, memory, and exhausted T cells, through the regulation of mTOR. A common viral strategy involves promoting the proliferation of Tregs while inhibiting the function of effector T cells (Zeng and Chi, 2017). For example, EBV encodes proteins that activate the PI3K Akt signaling pathway. This activation drives the significant expansion of Tregs, enhancing their immunosuppressive effects, while simultaneously inhibiting T cell receptor (TCR)-mediated proliferation signals. Consequently, effector T cells are rendered unable to effectively recognize and clear EBV-infected targets (Yuan et al., 2025). Furthermore, mTORC2 plays a critical role in humoral immunity by integrating TCR signaling and ICOS co-stimulation. This integration promotes the late differentiation and functional maturation of virus-specific Follicular Helper T cells (Tfh), which are essential for B cell-mediated germinal center reactions (Hao et al., 2018).

In summary, these findings demonstrate mTOR signaling inhibition as a pivotal mechanism for viral immune evasion, effectively coupling the regulation of protein synthesis and autophagy with the suppression of antiviral signaling. This highly context-dependent manipulation underscores the dualistic role of mTOR as both a driver of viral replication and a critical regulator of host defense.

## 5 The role of mTOR in antiviral immunity

The mTOR pathway serves not only as a central metabolic regulatory hub targeted by viruses but also as a crucial component of the host's antiviral immune defenses (Ye et al., 2017). By modulating the activation of innate immunity and orchestrating the differentiation and functional maintenance of adaptive immune cells, mTOR establishes a multi-layered and integrated antiviral immune network (Fekete et al., 2020; Chen et al., 2023b; Patel and Powell, 2025). Specifically, mTORC1 drives the differentiation of effector immune cells and manages acute-phase immune responses (Fekete et al., 2020), while mTORC2 plays a critical role in immune cell memory formation and long-term functional maintenance (Wang et al., 2022b). Together, mTOR signaling constitutes a critical immunometabolic axis that underpins effective antiviral defense.

### 5.1 The regulatory role of mTOR in innate immunity

Rapid metabolic reprogramming is essential for the innate immune system to support cell migration, proliferation, and the efficient production of cytokines (Weichhart et al., 2015; Møller et al., 2022). As key sentinels of innate immunity, DCs critically depend on mTOR activity to integrate TLR signaling with nutrient sensing, thereby regulating glycolysis, protein synthesis, cytokine secretion, and antigen processing. Inhibition of mTORC1 can enhance autophagy in DCs, may promote cross-presentation and can bias DCs toward a less immunogenic or regulatory phenotype (Snyder and Amiel, 2019; Sukhbaatar et al., 2016). Viral modulation of mTOR disrupts these processes, impairing DC maturation and weakening the bridge between innate and adaptive immunity. For example, HIV-1 activates mTOR in DCs to rapidly inhibit autophagy, thereby enhancing viral retention while concurrently impairing pathogen degradation, TLR signaling, antigen presentation, and the initiation of innate–adaptive immune responses (Blanchet et al., 2010). mTORC2 has emerged as a pivotal role in regulating antiviral innate immune responses. In HSV-1 infection, mTORC2-mediated AKT activation suppresses pro-apoptotic signaling while supporting effective immune responses, thereby promoting cell survival and host protection (Suryawanshi et al., 2021). Loss of mTORC2 signaling results in impaired immune infiltration and increased viral burden, underscoring its protective role.

In addition, mTOR governs monocyte and macrophage development and IFN signaling networks. Evidence suggests that mTOR deficiency impairs monocyte maturation through dysregulated STAT5–IRF8 signaling (Zhao et al., 2018). Furthermore, mTOR signaling is tightly associated with the antiviral IFN cascades. In porcine alveolar macrophages infected with PRRSV, the mTOR pathway forms a bidirectional loop with type I IFN signaling that governs antiviral gene expression. Specifically, mTOR signaling regulates PRRSV infection, at least in part, by modulating the production and signaling of type I IFNs (Liu et al., 2017a).

During viral infection, Natural Killer (NK) cells are activated primarily by cytokine signaling, such as IL-12, IL-15, and IL-18, in conjunction with signals from NK cell receptors, triggering metabolic reprogramming (Fallone et al., 2025; Terrén et al., 2021). This process enhances glycolysis and oxidative phosphorylation (OXPHOS) to meet the energetic and biosynthetic demands for cytotoxic activity, cytokine secretion, and proliferation (Fallone et al., 2025; Walls et al., 2020). mTORC1, acting alongside other metabolic regulators, serves as a critical control point for these adaptations in NK cells responding to viral challenges (Donnelly et al., 2014). Current evidence indicates that NK cell metabolism, mediated by mTOR-dependent pathways, significantly influences effector functions during acute, chronic, or reactivation phases of infection. In summary, mTOR integrates metabolic adaptation with innate immune activation, enabling rapid and effective frontline antiviral responses.

### 5.2 The regulatory role of mTOR in adaptive immunity

Within adaptive immunity, the mTOR pathway orchestrates the fate and functional specialization of T and B cells (Ma et al., 2024). In T cells, elevated mTORC1 activity typically promotes the differentiation of naïve T cells into effector lineages, including Th1, Th17, and cytotoxic CD8^+^ subsets, which are central to eliminating virus-infected cells (Ma et al., 2024). Metabolic analyses have emphasized that mTOR-dependent metabolic programs, particularly glycolysis, are central to CD8^+^ T-cell effector differentiation and antiviral function. Elevated glycolytic activity provides the energetic and biosynthetic resources necessary for rapid proliferation and cytotoxicity. In chronic infections like HIV, impaired metabolic fitness and dysregulated glycolytic capacity are associated with diminished effector function and immune imbalance (Rahman et al., 2021; Chen et al., 2023b). Conversely, diminished mTOR activity and reduced glycolysis favor the expansion of Tregs, thereby maintaining immune tolerance and hindering effective viral clearance (Procaccini and Matarese, 2012). This differentiation bias is closely linked to the regulation of fatty acid oxidation (FAO) following mTOR inhibition, a metabolic pathway that provides sustained energy support required for Treg function (Lu et al., 2025).

The regulation of T cell fate by mTORC2 primarily governs the establishment and maintenance of memory functions (Chen et al., 2023b). Research has demonstrated that mTORC2 signaling, partially via AKT phosphorylation at Ser473, supports the metabolic remodeling associated with memory differentiation, including a shift towards oxidative metabolism (Gabriel et al., 2021; Chen et al., 2023b). This metabolic switch is essential for the long-term survival and rapid responsiveness of memory CD8^+^ T cells (Chen et al., 2023b). Indeed, following acute viral infection, mTORC2-deficient (Rictor−/−) mice exhibit a significantly reduced number of virus-specific memory CD8^+^ T cells, resulting in impaired viral clearance upon secondary challenge (Chen et al., 2023b; Pollizzi et al., 2015).

In the context of chronic viral infections—such as HIV and HCV—pathogens manipulate these mTOR-dependent pathways to promote persistence and subvert host defenses (Akbay et al., 2020; Shrivastava et al., 2012). Zheng et al. (2022) provide evidence that chronic viral infection induces metabolic dysfunction in effector T cells, including inhibition of the PI3K/AKT/mTORC1 axis, which contributes to progressive T-cell exhaustion and diminished antiviral capacity (Zheng et al., 2022). Clinical studies indicate altered metabolic signaling and increased Treg frequencies during chronic HIV infection; notably, this immune imbalance correlates positively with viral load (Presicce et al., 2011; Rahman et al., 2021). Mechanistically, because effector T cells rely heavily on mTOR-mediated glycolysis while Tregs depend primarily on FAO (Salmond, 2018), viruses exploit this metabolic divergence to further suppress antiviral immune responses (Magalhaes et al., 2019).

B cells rely on the mTOR pathway—engaging both mTORC1 and mTORC2—for critical processes such as germinal center (GC) formation, antibody affinity maturation, and memory cell generation. Recent investigations indicate that B cell specific deletion of Raptor markedly restricts GC B cell proliferation during acute viral infections, such as lymphocytic choriomeningitis virus (LCMV), consequently weakening virus-specific antibody production (Li et al., 2017). Indeed, mTOR signaling is essential for antiviral humoral immunity by coordinating B-cell-intrinsic activation and CD4^+^ T follicular helper (Tfh) cell responses; In the LCMV infection model, deficiency in mTOR signaling directly interferes with B cell receptor (BCR)-dependent, antigen-specific activation, while having minimal effect on lipopolysaccharide-induced activation (Ye et al., 2017). Mechanistically, mTORC2 regulates B-cell growth, metabolism, and function by activating AKT, which subsequently promotes mTORC1 activity (including S6K1 phosphorylation) and stabilizes c-Myc (Li et al., 2019a). Furthermore, mTORC1 plays a pivotal role in antibody class switching by modulating the 4E-BP/eIF4E translational axis (Chiu et al., 2019; Ripperger and Bhattacharya, 2021). Collectively, these findings underscore the central role of mTOR signaling in regulating humoral immune responses (Iperi et al., 2021).

Together, mTOR acts as a central immune-metabolic regulator that directs adaptive immune differentiation, memory formation, and antibody-mediated antiviral protection.

### 5.3 The dualistic roles of mTOR immune regulation

Notably, mTOR serves as a double-edged sword in immune regulation. While basal and appropriately regulated mTOR activity is essential for immune cell activation, effector differentiation, and viral clearance, its dysregulation can precipitate severe inflammatory tissue damage (Maiese, 2020). On the protective side, mTOR activity underpins the development of adaptive immunity, which is essential for viral clearance. Specifically, mTOR signaling shapes B cell and CD4^+^ T cell responses within germinal centers upon viral infection, thereby modulating the magnitude and quality of antibody production and cross-protective humoral immunity (Keating et al., 2013). Moreover, balanced upstream PI3K/AKT/mTOR signaling is critical for sustaining effector function and preventing dysfunction during acute viral infections, ensuring a durable antiviral response (Abu-Eid and Ward, 2021). Thus, maintaining baseline mTOR activation is critical for the effective suppression of viral invasion.

In acute infections like COVID-19, dysregulated inflammatory signaling, including mTOR, contributes to the excessive inflammatory responses, including cytokine storm and thrombotic complications, where the immune response causes more collateral damage than the virus itself. Mechanistically, mTOR promotes this inflammation by regulating the NLRP3 inflammasome, expansion of pro-inflammatory Th1/Th17 cells, and NF-κB-associated inflammatory pathways (Zhao et al., 2021; González-Dominguez et al., 2025; Perl, 2016; Li et al., 2019c). Experimental modulation of mTOR activity has been shown to attenuate these hyperinflammatory states, highlighting its therapeutic relevance (Toner et al., 2025). Therefore, antiviral strategies must aim for precise modulation of mTOR activity to balance effective viral clearance with the prevention of hyperinflammation.

Taken together, the above findings position mTOR as a master regulator that integrates metabolic reprogramming with immune function, coordinating innate immune activation, adaptive immune differentiation, and durable antiviral protection. The dualistic nature of mTOR underscores the necessity for precise control to achieve effective viral clearance while avoiding immune-mediated tissue damage.

## 6 mTOR-targeted antiviral strategies: potential, evidence, and optimization directions

As the mTOR pathway is central to both viral replication and antiviral immunity (Ando et al., 2023; Shives et al., 2016), mTOR-targeted therapeutic strategies have become a priority in antiviral research. The primary advantage of this approach lies in its ability to exert antiviral effects by regulating host signaling pathways, offering the potential for broad-spectrum efficacy against diverse viruses (Heredia et al., 2015; Ibrahim et al., 2022; Takeshita et al., 2012). However, the systemic nature of mTOR functions also poses a risk of adverse effects, and the ambiguous boundary between preclinical findings and clinical evidence may lead to misinterpretations regarding therapeutic application (Izzedine et al., 2013). Therefore, it is essential to clarify the stratification of evidence and optimize regulatory strategies to ensure the safe and effective development of these therapies.

### 6.1 Core advantages and overall challenges

The core advantage of mTOR-based antiviral approaches lies in their host-oriented mechanism: instead of directly targeting the virus, these strategies indirectly suppress viral replication by modulating host cell metabolic, autophagic, and survival signaling pathways (Yin et al., 2018). This approach reduces the likelihood of inducing viral drug-resistant mutations and offers the potential for broad-spectrum inhibition against diverse viruses dependent on the mTOR pathway (Gu et al., 2025; Meade et al., 2023; Yin et al., 2018). Furthermore, because mTOR governs both viral protein synthesis and host immune responses, targeting this pathway can exert a dual effect: suppressing viral replication while simultaneously modulating immune function (Itakura and McCarty, 2013). However, this strategy faces significant limitations. Since mTOR signaling is integral to fundamental physiological processes, including cell growth, metabolism, and survival, systemic suppression can lead to severe adverse effects, such as immunosuppression and metabolic disorders (Fraenkel et al., 2008). Moreover, a notable translational gap exists between preclinical studies and clinical practice. Due to the complexity of mTOR mechanisms and issues with clinical practice, as frequently observed in oncology trials, the therapeutic benefits observed in preclinical models are frequently not realized in human trials (Shu et al., 2025). Finally, the response to mTOR inhibitors can be heterogeneous depending on the stage of viral infection and the host's immune response status, thereby complicating the implementation of personalized therapeutic regimens (Ando et al., 2023).

### 6.2 Classification of inhibitors and evidence stratification

Based on their molecular targets and mechanisms of action, current mTOR-directed antiviral agents can be broadly classified into four categories, each exhibiting distinct differences in preclinical and clinical evidence bases, efficacy profiles, and limitations.

#### 6.2.1 Rapamycin and its analogs (rapalogs)

As classic selective inhibitors for mTORC1, rapamycin and its analogs, such as sirolimus and everolimus, possess the most substantial clinical evidence base (Saxton and Sabatini, 2017). Extensive clinical evidence from transplant cohorts demonstrates that rapalog-based regimens significantly reduce the incidence of HCMV infection compared with traditional CNI (calcineurin inhibitor) therapies, such as cyclosporine or tacrolimus, likely through restoration of host antiviral immune function rather than direct suppression of viral replication (Benjamin et al., 2011; Bollano et al., 2024; Kaminski et al., 2022; Wolf et al., 2022).

Beyond HCMV, the antiviral effects of rapalogs exhibit genotype-specific variability. In HCV infection, mTOR inhibition suppresses replication of genotypes 2a and 3a, yet unexpectedly promotes genotype 1b replication, a phenomenon mechanistically linked to selective dependence on mTORC1 signaling components such as Raptor rather than mTORC2 (Frey et al., 2017; Stöhr et al., 2016). Preclinical studies further demonstrated potential inhibitory effects against various other viruses, such as Zika virus (ZIKV) (Sahoo et al., 2020), although clinical validation remains lacking. Despite antiviral potential, this class of inhibitors faces several critical limitations, including impairment of host intrinsic antiviral defenses, chronic metabolic toxicity, and restricted regulatory approval primarily for oncology and transplantation rather than antiviral therapy (Motzer et al., 2015; Sadowski et al., 2016; Shi et al., 2018; Yao et al., 2011).

#### 6.2.2 ATP-competitive mTOR inhibitors

ATP-competitive inhibitors, such as Torin-1, simultaneously inhibit phosphorylation of mTORC1 and mTORC2, demonstrating broader and more complete pathway inhibition in preclinical models compared to rapalogs (Moorman and Shenk, 2010).

Preclinical evidence strongly supports the antiviral potential of this inhibitor class. *In vitro* and animal studies demonstrate robust suppression of flavivirus replication, including ZIKV and DENV, particularly in viral contexts resistant to partial mTORC1 inhibition (Carter et al., 2022; Sahoo et al., 2020). Similar antiviral activity has been observed in coronaviruses, align with large-scale omics evidence revealing extensive viral hijacking of host translational machinery regulated by mTOR signaling (Zhang et al., 2022; Gordon et al., 2020).

However, clinical translation of ATP-Competitive Inhibitors faces significant challenges and remains constrained by substantial dose-limiting cytotoxicity, adaptive resistance mechanisms involving AKT reactivation independently of mTORC2, and long-term metabolic and immunological safety concerns (Chen et al., 2015; Limon et al., 2014; Limon et al., 2014, 2014; Liu and Sabatini, 2020; Rodrik-Outmezguine et al., 2011). Current efforts aim to develop next-generation inhibitors that achieve more precise inhibition while minimizing these toxic effects (Schram et al., 2025).

#### 6.2.3 PI3K/mTOR dual-target inhibitors

PI3K/mTOR dual-targeted inhibitors, including BEZ235, BGT226, Gedatolisib, simultaneously block upstream phosphatidylinositol 3-kinase (PI3K) and the mTOR kinase, attenuating PI3K–AKT–mTOR signaling (Rossetti et al., 2024; Santiskulvong et al., 2011; Wong et al., 2011; Fokas et al., 2012; Wang et al., 2013b).

Preclinical oncogenic virus–associated models provide strong mechanistic support for this strategy. In KSHV-driven primary effusion lymphoma (PEL), dual inhibitors induce apoptosis and suppress tumor growth in xenograft systems, underscoring the dependence of virus-transformed cells on PI3K/mTOR signaling (Bhatt et al., 2010). Preclinical antiviral studies further indicate direct inhibitory activity across multiple viral infections, including reduced IAV replication and improved survival in infected mice, suppressed viral protein production in rotavirus models, and *in vitro* suppression of SARS-CoV-2 replication (Abu-Eid and Ward, 2021; Acharya et al., 2022; Smallwood et al., 2017; Yin et al., 2018).

Clinical evidence indicates that a strategy combining low-dose BEZ235 plus RAD001 enhanced antiviral gene expression, improved IAV vaccine responses, and significantly reduced infection rates in elderly adults, demonstrating clear host-directed antiviral potential (Mannick et al., 2018). However, outside this specific low-dose immunomodulatory context, direct clinical antiviral efficacy of PI3K/mTOR dual inhibitors remains unestablished and largely extrapolated from preclinical infection and oncology models (Fattahi et al., 2022). Further *in vitro* and *in vivo* investigations therefore remain essential to define therapeutic windows, antiviral potency, toxicity profiles, and pharmacokinetic/pharmacodynamic evaluations across diverse viral systems before broader clinical translation can be considered.

#### 6.2.4 Inhibitors of downstream effector molecules

Targeting downstream effectors of mTOR, such as p70S6K1 and 4E-BP1 translational axis, offers a mechanistically attractive route to selectively suppress virus-dependent protein synthesis while potentially sparing many of mTOR's core roles in cell survival and metabolism (Dowling et al., 2010; Ohanna et al., 2005).

Preclinical studies indicate that selective S6K1 inhibitor PF-4708671 effectively blocks downstream phosphorylation *in vitro*, but exhibits inconsistent antiviral efficacy, including failure to suppress viral protein production in IAV models, suggesting that the antiviral activity of S6K1 inhibitors may be highly context-dependent, influenced by viral strain, cell type, and experimental conditions (Pearce et al., 2010; Wang et al., 2020). Moreover, mTOR inhibition can activate compensatory translation pathways, such as PI3K/MnK-mediated eIF4E hyperphosphorylation, paradoxically enhancing viral protein synthesis in CHIKV infection (Joubert et al., 2015). Modulation of the 4E-BP/eIF4E axis can be implicated in regulating viral latency in specialized cellular systems, by influencing translation of specific subsets of host and viral mRNAs (Kobayashi et al., 2012).

In summary, selective inhibitors of S6K1 or modulators of the 4E-BP1/eIF4E axis remain promising preclinical tools to dissect virus–host translation dependencies and to explore more narrowly targeted antiviral strategies within the mTOR signaling network (Kobayashi et al., 2012; Wang et al., 2020). However, current clinical evidence does not yet support broad claims of high-efficacy antiviral activity for these downstream effector inhibitors, highlighting the need for systematic comparative studies across diverse viral models and physiologically relevant cell systems.

### 6.3 Optimization strategies: from single targeted inhibition to synergistic regulation

To address the limitations of existing inhibitors, it is essential to shift from single targeted inhibition to multi-dimensional synergistic regulation.

#### 6.3.1 Precise application of host-directed therapy

The essence of host-directed therapy (HDT) lies in precise regulation rather than complete pathway shutdown, with the goal of blocking viral metabolic support while preserving essential host immune functions (Terrazzano et al., 2020). In the context of viral infections, low-dose mTOR inhibition has been shown to enhance antiviral immune responses by increasing ISG expression, while minimizing the immunosuppressive side effects observed at high doses (Mannick et al., 2018). These findings indicate that carefully timed, partial modulation of mTOR activity can enhance antiviral immunity rather than merely inhibiting cellular growth or metabolism. This concept is further supported by clinical evidence from other infectious settings, where low-dose everolimus as an adjunctive HDT was demonstrated to be safe and associated with improved lung function recovery in tuberculosis patients (Wallis et al., 2021).

#### 6.3.2 Combination therapy strategies

Combination therapy represents a crucial strategy for enhancing efficacy and reducing side effects. Rather than single node treatment, rational combinations involving mTOR inhibitors can amplify antiviral or immunomodulatory outcomes across diverse disease contexts. Combinations of mTOR inhibitors with immunomodulators, such as PD-1 blockade, have demonstrated synergistic benefits by suppressing PI3K–AKT–mTOR signaling, reducing immune-related toxicities, and enhancing immune effector function, and improving the safety window for checkpoint inhibition (Bai et al., 2021). In viral infections, for instance, pairing mTOR inhibitors with direct-acting antivirals, exemplified by sirolimus combined with maribavir in HCMV treatment, potentiates pronounced antiviral efficacy by limiting cellular metabolic compensation mechanisms. (Chou et al., 2018). Synergistic effects have also been observed with other host-targeted agents, such as chloroquine, through concurrent disruption of autophagy-mediated survival and mTOR-driven proliferative signaling (Knigin et al., 2021).

#### 6.3.3 Phased medication regimens

Given the dynamic progressions of viral infections, effective application of mTOR inhibitors requires phase-specific therapeutic strategies. During early infection stages characterized by T cell expansion, transient mTOR blockade enhances antiviral immunity by promoting the accumulation of stem-like T cells. In contrast, sustained or late-stage mTOR inhibition may impair antiviral effector responses in certain contexts (Ando et al., 2023). Drug-specific pharmacodynamic profiles further influence therapeutic outcomes. ATP-competitive inhibitors exhibit rapid antiviral activity accompanied by enhanced type I IFN responses, whereas rapamycin requires prolonged exposure to achieve comparable antiviral effects, likely reflecting differences in the kinetics and completeness of mTOR pathway suppression (Liu et al., 2017a). Together, these observations underscore the importance of temporal precision and drug-specific properties in optimizing mTOR-targeted antiviral regimens.

### 6.4 Challenges and prospects

Although mTOR-targeted antiviral strategies exhibit considerable therapeutic potential, several major challenges continue to limit their clinical translation. (1) Lack of tissue specificity: Systemic administration of these drugs could adversely impact the metabolism and function of normal tissues (e.g., liver, kidneys, and hematopoietic system) (Augustine et al., 2004; Huber et al., 2011; Sung et al., 2021). Consequently, the development of delivery technologies, such as liposomes and viral vectors, is required to specifically target infected tissues, thereby maximizing local drug concentration while minimizing systemic side effects (Li et al., 2021). (2) Heterogeneity in patient response: Therapeutic efficacy varies significantly among individuals due to differences in immune response, metabolism, and stage of the viral infection (Ando et al., 2023; Sun et al., 2023). Integrating multi-omics data (including transcriptomics and metabolomics) to build predictive models will be crucial for guiding personalized medication (Rashid and Selvarajoo, 2024). (3) Long-term safety and drug resistance: Prolonged use of mTOR inhibitors may lead to chronic adverse effects, such as metabolic disorders and immune dysfunctions (Kezic et al., 2018).

To address these hurdles, future research should focus on three key directions: Firstly, sophisticated tools such as single-cell omics and spatial transcriptomics should be employed to thoroughly analyze the spatiotemporal dynamics of mTOR pathway activity across specific cell types during various stages of viral infection (Virgin et al., 2009; Thaker et al., 2019). Second, increasing attention must be paid to develop inhibitors with high specificity for individual mTORC1/mTORC2 subtypes, as well as agents with cell type–restricted activity. For instance, nanoparticle-mediated delivery strategies have successfully selectively suppressed mTORC2 signaling in oncology models, illustrating the feasibility of cell type–specific pathway targeting. Third, multidimensional combination approaches that integrate “mTOR regulation, immunotherapy, and directed delivery” should be explored to develop broad-spectrum, efficient, and low-toxicity antiviral therapies (Werfel et al., 2018).

## 7 Summary and prospects

The mTOR pathway acts as a central signaling axis in virus-host interactions, integrating the metabolic and immunological signals that govern viral infection dynamics. Viruses have evolved diverse strategies to regulate the mTOR pathway, tailored to their specific replication characteristics and pathogenic requirements. DNA viruses, such as HCMV, HBV, and EBV, typically sustain mTOR activation to drive metabolic predation. In contrast, RNA viruses like DENV and ZIKV often exploit cellular autophagy through inhibitory or uncoupling strategies. Emerging viruses, such as SARS-CoV-2, exhibit a unique pattern of activating mTORC1 while simultaneously blocking autophagy, whereas chronic viruses like HIV and HCV maintain a balance between replication and immune evasion through bidirectional regulation. Host cells counteract these tactics by leveraging mTOR-driven metabolic defense and immune clearance mechanisms; ultimately, this dynamic regulatory balance determines the outcome of infections.

While existing studies have illuminated the core role of the mTOR pathway in viral infections, several key scientific questions remain. These include the functional specificity and synergistic mechanisms of mTORC1 and mTORC2 across various infections; novel molecular targets for viral regulation of mTOR; crosstalk mechanisms between mTOR pathway and the IFN or inflammasome pathways; and tissue-specific differences in mTOR regulation by prominent viruses like HBV and SARS-CoV-2. In-depth exploration of these issues will provide fresh insight into the molecular basis of virus-host interactions.

Future research should focus on four key directions. First, employing single-cell omics, spatial transcriptomics, and structural biology to accurately map the spatiotemporal dynamics of mTOR pathway activity and its crosstalk with other metabolic or immune signaling pathways during viral infection. Second, developing highly selective, cell-type-specific mTOR regulatory tools—such as liver-targeted inhibitor delivery systems, mTORC2-specific activators, and monocyte-macrophage-targeted mTOR modulators—will provide more precise therapeutic options. Third, exploring combination strategies that integrate mTOR-targeted therapy with immunotherapy and direct-acting antivirals: for example, co-administering mTOR inhibitors with PD-1 inhibitors for chronic HIV or with IFN-α for DENV could enhance efficacy and minimize side effects through synergistic mechanisms. Finally, leveraging AI-driven modeling and network analysis to predict mTOR-driven metabolic states that favor viral infection, uncover novel therapeutic targets, and integrate multi-omics data for personalized antiviral strategies. As our understanding of the mTOR signaling network and virus-host interactions deepens, mTOR-targeted strategies hold great promise for overcoming existing limitations. These approaches have the potential to evolve into novel, broad-spectrum, efficient, and low-toxicity therapies, offering critical solutions for emerging and re-emerging viral infections.

## CRediT authorship contribution statement

**Zizhen Ming:** Writing – review & editing, Writing – original draft, Formal analysis. **Bing Su:** Writing – review & editing, Supervision. **Qiming Liang:** Writing – review & editing, Writing – original draft, Supervision, Formal analysis.

## Declaration of competing interest

The authors declare that they have no known competing financial interests or personal relationships that could have appeared to influence the work reported in this paper.
